# Effective control of neuropathic pain by transient expression of hepatocyte growth factor in a mouse chronic constriction injury model

**DOI:** 10.1096/fj.201800476R

**Published:** 2018-04-16

**Authors:** Boram Nho, Junghun Lee, Junsub Lee, Kyeong Ryang Ko, Sung Joong Lee, Sunyoung Kim

**Affiliations:** * School of Biological Sciences, Seoul National University, Seoul, Korea;; † ViroMed, Seoul, South Korea;; ‡ Department of Neuroscience and Physiology, Seoul National University, Seoul, South Korea

**Keywords:** VM202, plasmid DNA, gene therapy, DRG, microglia

## Abstract

Hepatocyte growth factor (HGF) is a multifunctional protein that contains angiogenic and neurotrophic properties. In the current study, we investigated the analgesic effects of HGF by using a plasmid DNA that was designed to express 2 isoforms of human HGF—pCK-HGF-X7 (or VM202)—in a chronic constriction injury (CCI) –induced mouse neuropathic pain model. Intramuscular injection of pCK-HGF-X7 into proximal thigh muscle induced the expression of HGF in the muscle, sciatic nerve, and dorsal root ganglia (DRG). This gene transfer procedure significantly attenuated mechanical allodynia and thermal hyperalgesia after CCI. Injury-induced expression of activating transcription factor 3, calcium channel subunit α2δ1, and CSF1 in the ipsilateral DRG neurons was markedly down-regulated in the pCK-HGF-X7–treated group, which suggested that HGF might exert its analgesic effects by inhibiting pain-mediating genes in the sensory neurons. In addition, suppressed CSF1 expression in DRG neurons by pCK-HGF-X7 treatment was accompanied by a noticeable suppression of the nerve injury–induced glial cell activation in the spinal cord dorsal horn. Taken together, our data show that pCK-HGF-X7 attenuates nerve injury–induced neuropathic pain by inhibiting pain-related factors in DRG neurons and subsequent spinal cord glial activation, which suggests its therapeutic efficacy in the treatment of neuropathic pain.—Nho, B., Lee, J., Lee, J., Ko, K. R., Lee, S. J., Kim, S. Effective control of neuropathic pain by transient expression of hepatocyte growth factor in a mouse chronic constriction injury model.

Neuropathic pain is a pathologic chronic condition caused by dysfunction in the nervous system. Clinical symptoms of neuropathic pain include allodynia, pain sensation in response to non-noxious stimuli, and hyperalgesia—an increased sensitivity to pain (1, 2). Millions of people worldwide suffer from this devastating disease, yet it remains a major clinical challenge because its pathologic mechanisms are poorly understood. Current treatment methods for neuropathic pain include anticonvulsants, antidepressants, and opioids (2, 3), each of which provides only limited efficacy that is palliative rather than curative and often causes significant adverse effects. Therefore, there is a great need to develop safer, more effective therapeutic drugs.

In an effort to develop an effective treatment method for painful neuropathy, we have been exploring the possibility of using plasmid expression human hepatocyte growth factor (HGF). HGF is a multifunctional protein with potent neurotrophic properties that are produced by various cell types of mesenchymal origin (4–8). In humans, 2 isoforms of HGF, namely HGF723 (or dHGF) and HGF728 (or cHGF), are produced from *HGF* by alternative splicing (9, 10). Coexpression of these 2 isoforms has been reported to be necessary for optimum biologic activity of HGF (11, 12). Biologic functions of HGF are mediated by its receptor, called c-Met, and are expressed in sensory neurons, Schwann cells, and smooth muscle cells, among others (13, 14), all of which play important roles in the function of peripheral nerves after pathologic insults. Indeed, it has been reported that HGF acts on sensory neurons to enhance their survivability and neurite outgrowth (15–20). Moreover, an intramuscular injection of plasmid DNA–expressing HGF has been shown to significantly reduce neuropathic pain symptoms in phase I and II clinical studies (21, 22).

In the current study, we investigated the effects of HGF—delivered in the form of plasmid DNA—on neuropathic pain by using a chronic constriction injury (CCI) mouse model. pCK-HGF-X7 (or VM202) is a plasmid expression vector that was designed to produce 2 isoforms of human HGF (hHGF) (12, 23), which has been known to work as efficiently as endogenous HGF in animal models (11, 20, 24, 25). Moreover, this plasmid DNA has been assessed in various clinical studies, including those for critical limb ischemia, coronary artery disease, amyotrophic lateral sclerosis, and diabetic peripheral neuropathy, as well as in different animal models (11, 22, 26–34).

On the basis of these clinical studies, we have investigated the putative analgesic effects of pCK-HGF-X7 and its underlying mechanisms in a CCI-induced mouse neuropathic pain model. We found that an intramuscular injection of pCK-HGF-X7 effectively suppressed neuropathic pain for several weeks in mice after CCI, and also inhibited the expression of various genes that are involved in nerve injury and neuropathic pain development. Of particular importance, pCK-HGF-X7 injection inhibited nerve injury–induced CSF1 expression in injured dorsal root ganglions (DRGs) and subsequent microglia and astrocyte activation in the spinal cord. Taken together, our data suggest that an intramuscular injection of pCK-HGF-X7 could effectively reduce neuropathic pain by modulating the expression of injury- or pain-related factors, thereby controlling the complex pain transmission circuitry.

## MATERIALS AND METHODS

### Animals and surgery

Male Imprinting Control Region (ICR) mice (24–26 g) were purchased from Orient Bio (Seoul, South Korea) and used for behavioral studies. All surgical and experimental procedures were approved by the Institutional Animal Care and Use Committee at Seoul National University. Animals were housed in the animal facility for at least 7 d under a 12-h light/dark cycle before receiving any surgeries or treatments. Mice had access to food and water *ad libitum*.

Mice were anesthetized with an injection of alfaxan (80 mg/kg, i.p.). Neuropathic pain was mimicked by using CCI of the sciatic nerve (35), which consists of several processes that result in chronic nerve injury in the periphery. To perform CCI, mice underwent ∼1-cm-long blunt dissections to expose the right sciatic nerve, which usually lies between the gluteal and biceps femoris muscles. Once the sciatic nerve proximal to the trifurcation site was exposed, it was given loose ligatures 3 times with 0.5-mm spacing using 6-0 silk (Ailee, Busan, South Korea) sutures. Ligatures were slightly tightened until there was a noticeable twitch of the right hind limb. One hundred microliters control plasmid, pCK (2 mg/ml), or pCK-HGF-X7 (2 mg/ml) were then injected intramuscularly in the area of the bicep femoris and quadriceps femoris muscles. Sham-treated mice received the same dissections in the right thigh, but did not receive ligatures in the sciatic nerve. As a control, sham-treated mice were injected intramuscularly with 50 μl of filtered PBS into the same muscles. After surgery, lesions were closed with sutures and povidone-iodine was applied to prevent wound infection. Animals had ointment applied to their eyes to prevent corneal drying and damage from anesthetics, and were monitored under a warm pad during recovery.

### Plasmid DNA preparation

Cloning of genomic–cDNA hybrids of *HGF* and construction of expression vector pCK-HGF-X7 plasmid have been described in detail by Pyun *et al.* (12). All expression vectors used in experiments were purified by using an EndoFree plasmid Maxi or Giga Prep Kit (Qiagen, Hilden, Germany) dissolved in 0.9% NaCl, diluted to 2 mg/ml, and stored at −80°C before use.

### RNA analysis

For quantitative real-time RT-PCR, ipsilateral L4–L6 DRGs and L4–L5 spinal cords were collected 4 d after CCI. Samples were mechanically homogenized by using polypropylene pestles (Bel-Art Scienceware, Wayne, NJ, USA), and total RNA was extracted in RNAiso Plus (Takara, Kyoto, Japan). Quantification of RNA was performed by using a nanodrop instrument. Equal amounts of RNA were used to synthesize cDNAs by using Reverse-Transcriptase XL (AMV; Takara). Quantitative RT-PCR was performed by using SYBR Premix Ex Taq (Takara). Primers used for expression analysis were as follows: *ATF3* (activating transcription factor 3): forward 5′-GAGGATTTTGCTAACCTGACACC-3′, reverse 5′-TTGACGGTAACTGACTCCAGC-3′; *CACNA2D1*: forward 5′-CTGCTGGCCTTGACTCTGAC-3′, reverse 5′-CACTCCACTTGCTGTTTTTGC-3′; *CSF1*: forward 5′-TGCTAAGTGCTCTAGCCGAG-3′, reverse 5′-CCCCCAACAGTCAGCAAGAC-3′; cathepsin S gene: forward 5′-CCATTGGGATCTCTGGAAGAAAA-3′, reverse 5′TCATGCCCACTTGGTAGGTAT-3′; *IRF8* (interferon regulatory factor 8): forward 5′-GGGCAGCGTGGGAACC-3′, reverse 5′-GCTTCCAGGGGATACGGAAC-3′; *IRF5*: forward 5′-TGGGGACAACACCATCTTCA-3′, reverse 5′-CTGGAAGTCACGGCTTTTGT-3′; *Iba1* (ionized calcium-binding adapter molecule 1): forward 5′-ATCAACAAGCAATTCCTCGATGA-3′, reverse 5′-CAGCATTCGCTTCAAGGACATA-3′; *HPRT* (hypoxanthine-guanine phosophoribosyltransferase): forward 5′-TCAGTCAACGGGGGACATAAA-3′, reverse 5′-GGGGCTGTACTGCTTAACCAG-3′; and *GAPDH* (glyceraldehyde 3-phosphate dehydrogenase): forward 5′-AGGTCATCCCAGAGCTGAACG-3′, reverse 5′-CACCCTGTTGCTGTAGCCGTA-3′. Gene expression levels were normalized by using housekeeping genes—HPRT and GAPDH—and relative expressions were compared between respective experimental controls.

### Western blot analysis

For immunoblotting, animals were sacrificed 4 d after CCI and ipsilateral L4–L6 DRG neurons and L5 spinal dorsal horns were collected, followed by lysis using RIPA buffer with protease and phosphatase inhibitor cocktail (Roche Diagnostics, Indianapolis, IN, USA). Equal amounts of protein were separated on 10% SDS polyacrylamide gels and transferred to a Western blot membrane (PVDF). Membranes were blocked with 1% bovine serum albumin BSA in Tris-buffered saline with Tween 20 (TBST; 10 mM Tris-HCl, pH 7.4, 0.9% NaCl, and 0.1% Tween 20) for 1 h and probed with primary Abs that were diluted in blocking solution at 4°C overnight. Primary Abs included ATF3 (1:1000; Santa Cruz Biotechnology, Dallas, TX, USA), CACNA2D1 (1:1000; Thermo Fisher Scientific, Waltham, MA, USA), and β-actin (1:5000; MilliporeSigma, St. Louis, MO, USA). After washing with TBST, membranes were incubated with horseradish peroxidase–conjugated goat anti-mouse or rabbit IgG secondary Ab (MilliporeSigma) at room temperature for 1 h. Blots were then washed 3 times with TBST, and the protein bands were visualized using an ECL system (EMD Millipore, Billerica, MA, USA). Quantification of the bands was performed by using ImageJ software (National Institutes of Health, Bethesda, MD, USA). When needed, blots were stripped by using stripping buffer with vigorous shaking for 30 min at room temperature followed by 3 washes in TBST.

### ELISA

*In vivo* samples, including L4–L5 spinal cords, L4–L6 DRGs, sciatic nerves, and muscles, were prepared after CCI and homogenized in lysis buffer that contained protease inhibitor (11697498001; MilliporeSigma), phosphatase inhibitor cocktail (4906845001; MilliporeSigma), and PMSF (P-7626; MilliporeSigma) using polypropylene pestles (13-717-270; Thermo Fisher Scientific). Samples were centrifuged at 12,000 rpm for 15 min at 4°C, and supernatants that contained total protein were subjected to hHGF ELISA (SHG00; R&D Systems, Minneapolis, MN, USA) and mouse CSF1 ELISA (MMC00; R&D Systems) according to the manufacturer’s protocol. The level of HGF or CSF1 protein detected was normalized to the total amount of protein extracted from the tissue, as measured by a BCA Protein Assay Kit (Thermo Fisher Scientific).

### Immunofluorescence assay

Animals were deeply anesthetized, and we performed transcardial perfusion using PBS and 4% paraformaldehyde. Tissues of interest, including L4–L6 DRGs and L4–L5 spinal cords, were then removed and postfixed with 4% paraformaldehyde once again overnight at 4°C. Samples were washed with PBS once, followed by incubation in 30% sucrose phosphate buffer for 48 h. DRGs and spinal cord samples were embedded in optimum cutting temperature compound blocks (Tissue-Tek, Alphen aan den Rijn, The Netherlands) and sections were prepared on gelatin-coated glass slides using cryocut microtome (CM3050S; Leica, Wetzlar, Germany). Sections were blocked with 5% normal goat serum (Jackson ImmunoResearch Laboratories, West Grove, PA, USA), 2% bovine serum albumin, and 0.1% Triton X-100 (MilliporeSigma) that was dissolved in PBS for 1 h at room temperature. Samples were then washed 3 times with PBS and incubated overnight at 4°C with primary Abs. Primary Abs used in this study included Iba-1 (1:1000; Wako, Richmond, VA, USA) and glial fibrillary acidic protein (1:5000; Dako, Carpinteria, CA, USA). After rinsing with PBS, sections were incubated in secondary Abs for 1 h at room temperature. Secondary Abs used included donkey anti-mouse IgG Alexa Fluor 488 conjugate (1:1000; Thermo Fisher Scientific), donkey anti-rabbit IgG Alexa Fluor 488 conjugate (1:1000; Thermo Fisher Scientific), donkey anti-mouse IgG Alexa Fluor 555 conjugate (1:1000; Thermo Fisher Scientific), and donkey anti-rabbit IgG Alexa Fluor 555 conjugate (1:1000; Thermo Fisher Scientific). Thereafter, another 3 washes were performed using PBS, and nuclear staining was performed by using 1 μg/ml Hoechst 33248 (H1399; Thermo Fisher Scientific). Sections were mounted by using Vectashield mounting solution (H100; Vector Laboratories, Burlingame, CA, USA), and fluorescence images were obtained by using a confocal microscope (LSM700; Carl Zeiss, Jena, Germany). Quantification of fluorescent signal intensities was performed by using ImageJ software.

### Behavioral studies

Animals were habituated to the testing environment for 3 d before behavioral studies. The development of mechanical allodynia and thermal hyperalgesia was assessed by using von Frey filaments assay and Hargreaves test, respectively, and pain symptoms were evaluated weekly. Von Frey measurements were made to evaluate the mechanical sensitivity of mice. First, animals were placed individually in the cylinder on top of the metal mesh floor for 3 h for adaptation. Mechanical sensitivity of mice was assessed by stimulating the hind paw with von Frey filaments of different thicknesses. We also measured the frequency of hind paw withdrawal by using a constant thickness (0.16 g) of the filaments. Hargreaves test was also performed in a similar manner to assess the thermal sensitivity of nerve-injured mice. In brief, animals were placed on top of the heat-controlled glass for adaptation, then the hind paw was stimulated by radiant heat. Latency time was measured until the hind paw was withdrawn upon heat stimulation.

### Statistical analysis

All values are presented as means ± sem from more than 2 independent experiments. Differences between values were determined by 1- or 2-way ANOVA followed by Tukey’s *post hoc* test or Bonferroni’s multiple comparison test, provided by GraphPad Prism (GraphPad Software, La Jolla, CA, USA).

## RESULTS

### Intramuscular injection of pCK-HGF-X7 produces HGF proteins in the muscle, sciatic nerve, and DRG

**Figure 1:**
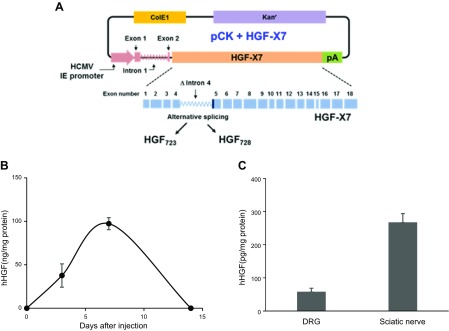
*A*) Diagram of pCK-HGF-X7 used in the study. The genomic–cDNA hybrid sequence of HGF is present in the pCK backbone as reported by Lee *et al*. (23). Major immediate–early (IE) region of HCMV (pink arrow, boxes, and wavy lines); HGF-X7 (orange box), which consists of genomic–cDNA hybrid sequence of HGF, including a truncated form of intron 4 (blue boxes and wavy lines); polyadenylation signal of bovine hormone gene (green box); the kanamycin resistant gene, phosphotransferase (purple box); and ColE1, the origin of replication, from *Escherichia coli* (yellow box). *B*) Intramuscular injection of pCK-HGF-X7 produces HGF proteins in the muscle, sciatic nerve, and DRG. Peripheral neuropathy was induced in 5-wk-old ICR mice by CCI, and 200 μg of pCK-HGF-X7 was injected intramuscularly on d 0. Time kinetics of hHGF expression. Skeletal muscles around the injection site were isolated at appropriate time points, and total proteins were prepared, followed by ELISA specific for hHGF (*n* = 4). *C*) hHGF expression in the peripheral nervous system. DRGs and sciatic nerves were isolated on d 4 after CCI and subjected to hHGF ELISA. In sham-treated and control vector lacking the HGF sequence (pCK) injected groups, hHGF was not detected (*n* = 4).

To investigate the effects of HGF on neuropathic pain, we used pCK-HGF-X7 (**Fig. 1*A***) in the current study. pCK-HGF-X7 is a 7377-bp-long plasmid DNA that has been used in previous studies of ischemic diseases (12, 23, 31–33). In addition to the usual replication origins (ColE1) and drug selectable marker (kanamycin resistance gene), it is composed of the 5′ control region from the immediate early region of human cytomegalovirus (including the promoter, exon 1, deleted intron 1, and untranslated exon 2), and genomic-complementary DNA hybrid sequence from *hHGF*. This gene consists of 18 exons and 17 introns spread over 67.4 kb, and the alternative splicing between exons 4 and 5 results in 2 isoforms of HGF—HGF723 and HGF728. The genomic–cDNA hybrid HGF sequence in pCK-HGF-X7 contains truncated intron 4, which was engineered to produce high levels of HGF723 and HGF728. Coexpression of these 2 isoforms was previously demonstrated to be biologically active in various studies using different animal models (11, 12).

To investigate the expression pattern of hHGF within muscles and the peripheral nervous system after pCK-HGF-X7 injection, we administered 200 μg of pCK-HGF-X7 to the thigh muscles using the mouse CCI model (35). To verify HGF protein expression derived from pCK-HGF-X7 around the injection site, total proteins were isolated from thigh muscles and the level of hHGF protein was measured by ELISA, which only detects hHGF and does not cross-react with endogenous murine HGF. The level of hHGF protein was gradually increased, reaching its highest point—97 ng/mg of total protein—at d 7, and thereafter decreased to an undetectable point by d 14 (Fig. 1*B*). Low, but readily detectable, levels of hHGF were reproducibly observed in the sciatic nerve (267 pg/mg) and DRG (58 pg/mg) 4 d after nerve injury (Fig. 1*C*). These results suggested that HGF produced from pCK-HGF-X7 in injected muscles could be delivered to peripheral nerve tissues and retrogradely transported to the DRG neurons. It has previously been shown that HGF produced from this plasmid does not circulate in a systemic manner, as it contains the heparin sulfate binding site in its N-terminal region (12, 38, 39).

### Intramuscular injection of pCK-HGF-X7 ameliorates nerve injury–induced neuropathic pain in a mouse CCI model

**Figure 2:**
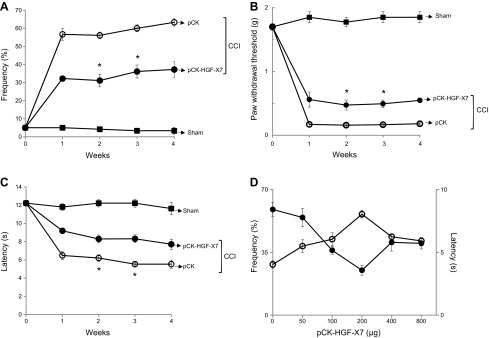
Intramuscular injection of pCK-HGF-X7 ameliorates nerve injury–induced neuropathic pain in a mouse CCI model. *A*–*C*) pCK and pCK-HGF-X7 (200 μg) were injected intramuscularly on the day of CCI, and the pain sensitivity toward mechanical and thermal stimuli was measured at appropriate times by von Frey filaments (*A*, *B*) and Hargreaves (*C*) tests, respectively. Each group consisted of 6 mice, and >3 independent experiments were performed (means + sem). Sham-treated mice (■); CCI + pCK (○); and CCI + pCK-HGF-X7 (●). *D*) Different doses of pCK-HGF-X7 were injected intramuscularly, and, 2 wk later, the pain sensitivity toward mechanical and thermal stimulation was measured by von Frey filaments (●) and Hargreaves (○) tests, respectively (*n* = 6/group; mean + sem). *P < 0.05, 2-way ANOVA.

We studied the effects of an intramuscular pCK-HGF-X7 injection on neuropathic pain development by measuring mechanical allodynia and thermal hyperalgesia by von Frey filaments assay and Hargreaves tests, respectively. Sham-treated mice were not affected in either test (**Fig. 2*A–C***). In CCI-induced and control vector (pCK) –injected mice, paw withdrawal frequency and threshold were changed in a way that was consistent with the increased level of mechanical allodynia (Fig. 2*A, B*). The level of thermal hyperalgesia was also increased, as evident by the reduction in thermal withdrawal latency (Fig. 2*C*); however, in animals that were injected with pCK-HGF-X7, the level of neuropathic pain was effectively suppressed, as demonstrated by a decrease in paw withdrawal frequencies or an increase in paw withdrawal thresholds and thermal withdrawal latencies (Fig. 2*A–C*). Pain-relieving effects lasted up to 4 wk with a single injection of pCK-HGF-X7 in this particular experiment and up to 8 wk in another longer-term experiment (Supplemental Fig. 1). These data show that a single intramuscular injection of pCK-HGF-X7 could reduce ongoing neuropathic pain for a long period of time without affecting baseline sensory function (Supplemental Fig. 2).

We investigated the effect of different doses of pCK-HGF-X7 in the context of pain relief. Five different concentrations of pCK-HGF-X7 were administered an intramuscular around the injury site on the day of ligation, and pain levels were measured at appropriate times by paw withdrawal frequency and thermal withdrawal latency. The behavioral test was measured 2 wk postinjury as that was the earliest time point at which a clear analgesic effect of pCK-HGF-X7 presents. As shown in Fig. 2*D*, the pain-relieving effect was increased in a dose-dependent manner, reaching a peak at 200 μg, then somewhat declining in both measurements. The optimum dose that produced the maximum pain-relieving effect was reproducibly found to be 200 μg of pCK-HGF-X7 in this particular model, and this concentration was used throughout the experiments.

### Repeated administration of pCK-HGF-X7 further attenuates neuropathic pain, depending on the time point of injection

**Figure 3:**
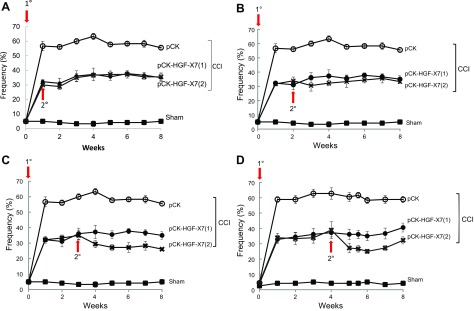
Repeated administration of pCK-HGF-X7 further attenuates neuropathic pain, depending on the time point of injection. pCK and pCK-HGF-X7 (200 μg) were delivered by intramuscular injection on the day of CCI, and the second injection was introduced, as indicated by the red arrows, after 1 (*A*), 2 (*B*), 3 (*C*), and 4 (*D*) wk. Pain sensitivity to a non-noxious mechanical stimuli was measured by von Frey filaments (*n* = 6/group; means + sem;). Sham-treated mice (■); CCI + pCK (○); CCI + pCK-HGF-X7 (1) (●); and CCI + pCK-HGF-X7 (2) (X).

We assessed whether repeated injection of pCK-HGF-X7 could further improve pain symptoms. When additional pCK-HGF-X7 injection was introduced at 1 or 2 wk after the initial injection, there was no significant influence in paw withdrawal frequencies (**Fig. 3*A, B***) compared with the group that received a single pCK-HGF-X7 injection; however, when the second pCK-HGF-X7 injection was introduced either 3 or 4 wk after the initial injection, the analgesic effect of pCK-HGF-X7 was further enhanced (Fig. 3*C*, *D*). These data suggest that the additional injection of pCK-HGF-X7 at a delayed time point could further improve the pain-relieving effect.

### Intramuscular injection of pCK-HGF-X7 attenuates neuropathic pain by down-regulating injury- and pain-related gene expression in DRG neurons

In the case of peripheral nerve injuries, the expression levels of various genes undergo extensive modifications in the DRG, eventually giving rise to pathologic conditions and high levels of pain (40). To understand the underlying mechanisms, we investigated the effects of pCK-HGF-X7 on the expression of several key injury- or pain-related genes.

**Figure 4:**
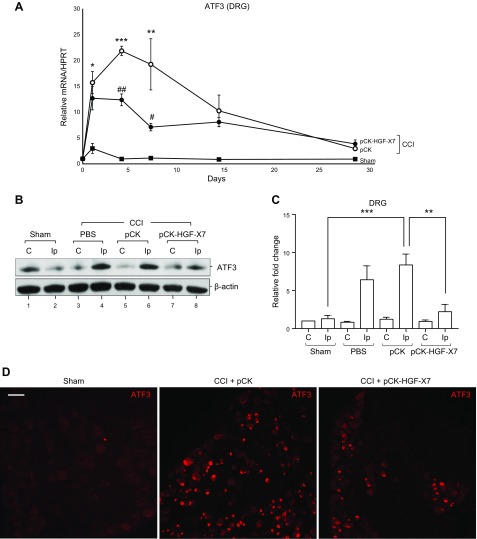
Intramuscular pCK-HGF-X7 injection attenuates ATF3 expression in DRG neurons. Intramuscular injection of 200 μg of pCK or pCK-HGF-X7 and CCI were introduced, and injured DRGs (L4–L6) were isolated 4 d later. Total RNAs, proteins, and DRG sections were prepared to perform quantitative RT-PCR (*A*), Western blot analysis (*B*, *C*), and immunostaining (*D*), respectively, using an Ab specific to ATF3. The level of mRNA was normalized to the expression of the contralateral DRG, and *HPRT* was used as housekeeping gene (*n* = 3/group; means + sem). Scale bar, 50 μm. In Western blot analysis, β-actin was used as a loading control. C, contralateral; Ip, ipsilateral. *P < 0.05, **P < 0.01, ***P < 0.001 compared with sham-treated group; ^#^*P* < 0.05, ^##^*P* < 0.01 compared with CCI + pCK group (1-way ANOVA).

ATF3 is a well-known marker for injured sensory neurons (41), and its level is known to be dramatically increased in the nucleus of DRG neurons upon peripheral nerve injury (42). We measured the RNA levels of ATF3 at various time points, including d 1, 4, 7, 14, and 28 after CCI (**Fig. 4*A***). In sham-treated animals, RNA level was slightly increased on d 1, presumably as a result of muscle incision, but returned to its basal level. In CCI-induced mice that were injected with pCK control, RNA level of ATF3 was up-regulated 22-fold, reaching a peak on d 4. When mice were administered pCK-HGF-X7, the ATF3 level was reduced by 44% (d 4) and 63% (d 7) compared with pCK control at a given time. By d 28, expression of ATF3 was close to its basal level in both pCK and pCK-HGF-X7–treated groups. To be certain, the protein level of ATF3 was also analyzed on d 4, which was found to be low in sham-treated animals (Fig. 4*B*, lane 2), but significantly increased in CCI-operated mice (Fig. 4*B*, compare lanes 2 and 4). ATF3 expression level of the control plasmid pCK-injected, CCI-operated mice was comparable to that of the PBS-injected, CCI-operated mice, which indicated that an intramuscular pCK injection, *per se*, had little effect on ATF3 expression (Fig. 4*B*, compare lanes 4 and 6); however, when mice were injected with pCK-HGF-X7, the injury-induced increase in ATF3 expression was markedly inhibited (Fig. 4*B*, compare lanes 6 and 8). Similar trends were also observed when ATF3 Western blot analysis was quantified by using ImageJ software (Fig. 4*C*). In an immunostaining assay, ATF3 was scarcely detected in sham-treated mice, but was highly increased in the L4–L5 DRG neurons of CCI-induced and pCK-injected mice (Fig. 4*D*). Treatment with pCK-HGF-X7 suppressed CCI-induced ATF3 expression 4 d after CCI (Fig. 4*D*). These results indicate that an intramuscular injection of pCK-HGF-X7 could protect DRG neurons from severe nerve damage, which might have halted or reversed the neuropathic pain and disease progression as observed in the previous experiments.

**Figure 5:**
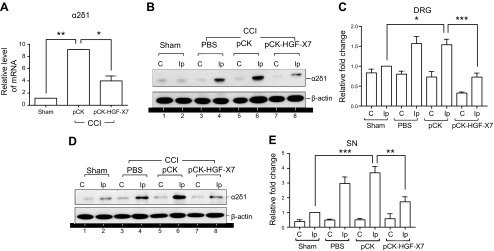
Intramuscular pCK-HGF-X7 injection attenuates α2δ1 expression in DRG neurons. *A*–*C*) Intramuscular injection of pCK or pCK-HGF-X7 (200 μg) and CCI were performed, and affected DRGs were prepared 4 d later for quantitative RT-PCR (*A*) and Western blot analysis (*B*, *C*). *D*, *E*) Similar procedures were performed for the analysis of sciatic nerve by Western Blot analysis. The level of mRNA was normalized to the expression of the contralateral DRG, and *HPRT* was used as housekeeping gene (*n* = 3/group; means + sem). In Western blot analysis, β-actin was used as a loading control (*n* = 3/group). C, contralateral; Ip, ipsilateral. *P < 0.05, **P < 0.01, ***P < 0.001 compared with sham-treated mice; 1-way ANOVA.

The calcium channel subunit α2δ1 is another factor known to be highly up-regulated in DRG neurons upon nerve injury and implicated in pain sensitization (43–46). Up-regulation of the α2δ1 subunit leads to an increase in calcium influx, which eventually facilitates the synaptic transmission of the pain circuits. We analyzed the expression pattern of α2δ1 by using quantitative real-time RT-PCR and Western blot in ipsilateral DRG neurons and sciatic nerves 4 d after CCI. The α2δ1 RNA level in injury-induced mice was increased 9-fold compared with that of sham-treated mice (**Fig. 5*A***); however, in pCK-HGF-X7–treated mice, α2δ1 level was decreased by 40–50%. To confirm the decreased expression of α2δ1 at the protein level, we performed Western blot analysis. The level of the α2δ1 protein was increased in the injured DRG and sciatic nerve upon CCI (Fig. 5*B*, *D*, compare lanes 2 with 4 or 6), but was decreased in pCK-HGH-X7–injected mice (Fig. 5*B, C*, lanes 6 and 8). Taken together, these results suggest that HGF produced from pCK-HGF-X7 might down-regulate α2δ1 expression and decrease the synaptic transmission of pain circuitry, which may, in part, contribute to the analgesic effects of pCK-HGF-X7.

**Figure 6:**
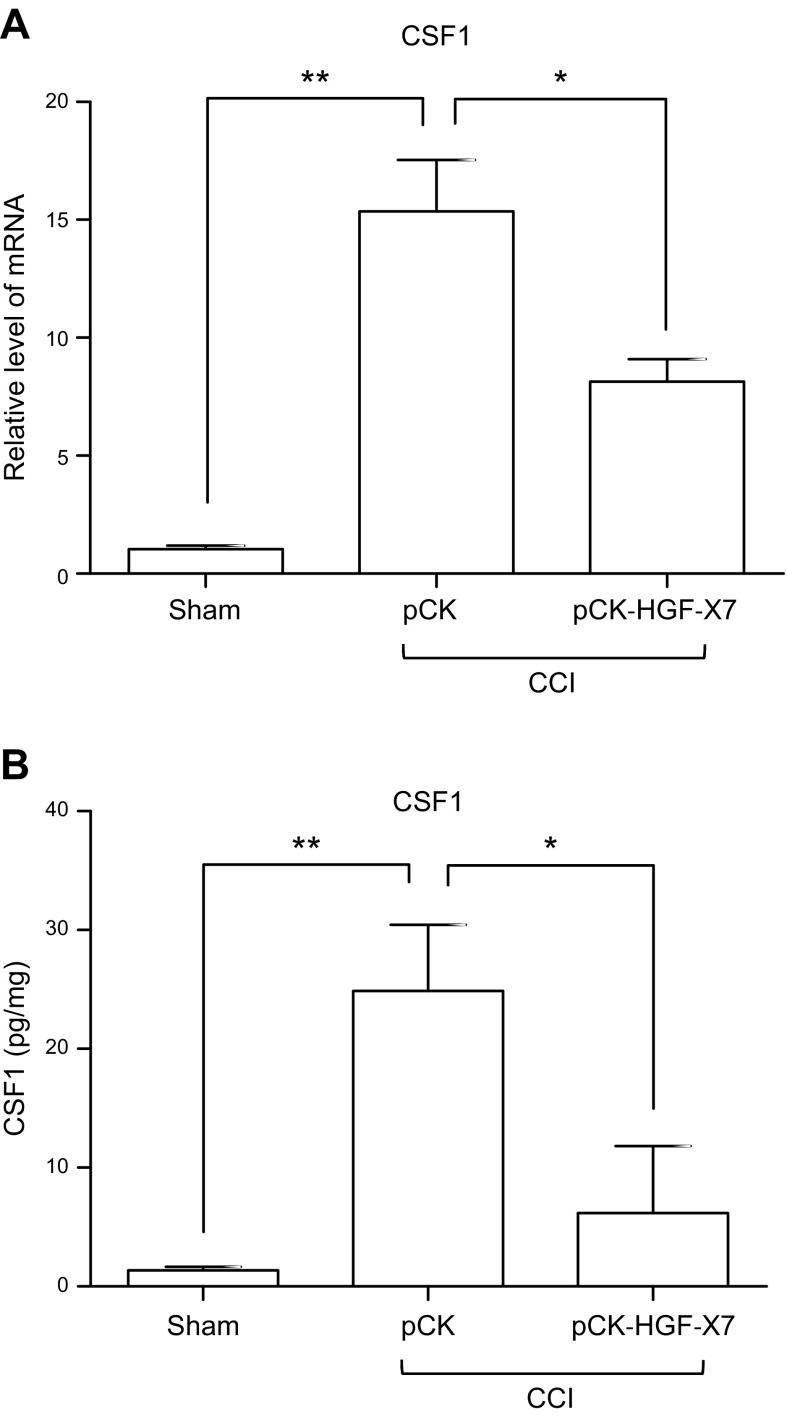
Intramuscular pCK-HGF-X7 injection attenuates CSF1 expression in DRG neurons. CCI was introduced, and 200 μg of pCK/pCK-HGF-X7 were transferred by intramuscular injection simultaneously. Ipsilateral DRGs (L4–L6) were isolated 4 d later. *A*, *B*) Total RNAs and proteins were prepared for quantitative RT-PCR (*A*) and ELISA (*B*) specific for CSF1, respectively. The level of mRNA was normalized to the expression of the contralateral DRG, and *HPRT* was used as housekeeping gene (*n* = 3/group; means + sem). *P < 0.05, **P < 0.01 compared with sham-treated mice; 1-way ANOVA.

CSF1 has recently been discovered to be a key factor that contributes to neuropathic pain by promoting the activation and proliferation of microglial cells in the spinal dorsal horn (47, 48). We measured the expression level of CSF1 in ipsilateral DRG 4 d after CCI. Quantitative RT-PCR analysis revealed that CSF1 was highly up-regulated ∼15-fold in injured sensory neurons. Intramuscular injection of pCK-HGF-X7 inhibited the CCI-mediated up-regulation of CSF1 by ∼50%, and similar levels of inhibition were observed when CSF1 protein levels were analyzed by ELISA (**Fig. 6*A, B***). These results suggest that HGF produced from pCK-HGF-X7 might exert its analgesic effects, at least in part, by down-regulating CSF1 expression in the DRG. Considering these data, it is conceivable that the effective control of neuropathic pain by intramuscular pCK-HGF-X7 injection might have resulted from modulating the expression of such key injury- or pain-related factors as ATF3, α2δ1, and CSF1.

### Nerve injury–induced spinal cord glial cell activation is attenuated by pCK-HGF-X7 injection

**Figure 7:**
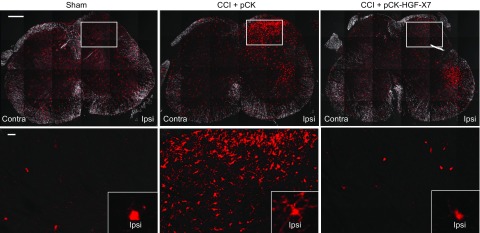
Nerve injury–induced spinal cord microglial activations are attenuated by pCK-HGF-X7 injection. CCI and intramuscular injection of 200 μg of pCK or pCK-HGF-X7 were performed. Affected spinal cords were removed 4 d later, and cryocut sections were prepared. Spinal cord sections were subjected to immunohistochemistry assay by using Abs that were specific to Iba1 (red; marker for microglia; *n* = 4). Scale bars, 200 (top row), 20 (bottom row) μm. Contra, contralateral; Ipsi, ipsilateral.

A major cause of neuropathic pain is known to be neuroinflammatory responses, which occur within the CNS, especially in the spinal cord dorsal horn (49–52). Several cell types that reside in the spinal cord, such as astrocytes and microglia, have been implicated in the development of central sensitization *via* complex modulation of the pain transmission circuitry (53). In particular, it was recently reported that CSF1 produced from injured DRG neurons could be transported to the dorsal horn spinal cord and induce microglial activation and proliferation, leading to the development of neuropathic pain and central sensitization (47, 48). To assess whether pCK-HGF-X7 plays a role in this pathway, we first investigated microglia activation in the spinal cord dorsal horn 4 d after CCI. As shown in **Fig. 7**, the density of microglia in sham-treated animals was comparable between contralateral and ipsilateral sides of the spinal cord; however, there was a substantial increase in the number of Iba1^+^ microglia in the ipsilateral dorsal horn after CCI. Furthermore, the morphology of the microglia that were present in the ipsilateral side was readily distinguishable from that of sham-treated mice. For example, in sham-treated mice, microglial cells seemed to be at the quiescent stage, whereas in the CCI-induced mice, Iba1^+^ cells were transformed into the activated morphology, with thicker processes (54); however, in the pCK-HGF-X7–treated group, the number of Iba1^+^ microglia was reduced, and the morphology was similar to that of microglia in their resting state. This result correlated well with the above data that show the inhibition of injury-mediated induction of CSF1 and pain reduction by pCK-HGF-X7.

**Figure 8:**
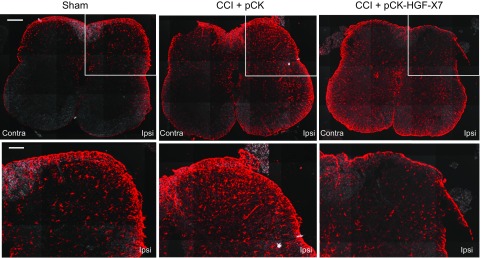
Nerve injury–induced spinal cord astrocyte activations are attenuated by pCK-HGF-X7 injection. CCI and intramuscular injection of 200 μg of pCK or pCK-HGF-X7 were performed. Affected spinal cords were appropriately prepared 4 d later, and cryocut sections were performed. Spinal cord sections were subjected to immunohistochemistry assay by using Abs that were specific to glial fibrillary acidic protein (GFAP; red; marker for astrocyte). Scale bars, 200 (top row), 100 (bottom row) μm. Contra, contralateral; Ipsi, ipsilateral.

As microglial activation is known to release ligands that also promote astrocyte activation (36, 54–56), we also assessed the effect of pCK-HGF-X7 on the profile of astrocytes. As shown in **Fig. 8**, CCI caused a marked increase in the number of glial fibrillary acidic protein–positive astrocytes on the ipsilateral side of the spinal cord dorsal horn. These changes were significantly attenuated in the pCK-HGF-X7–treated group.

**Figure 9:**
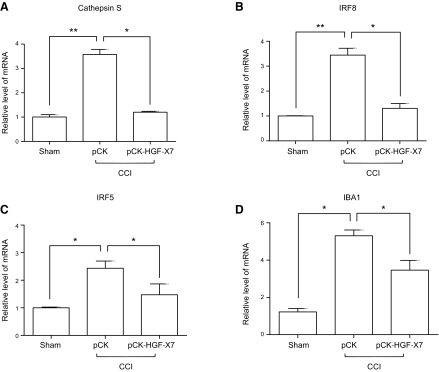
Nerve injury–induced spinal cord glial cell activations are attenuated by pCK-HGF-X7 injection. Four days after CCI and plasmid injection, the ipsilateral spinal cord dorsal horn was isolated and total RNAs were prepared, followed by quantitative RT-PCR analysis for respective genes. The level of mRNA of Cathepsin S (*A*), IRF8 (*B*), IRF5 (*C*), and IBA1 (*D*) was measured. They were normalized against GAPDH (glyceraldehyde 3-phosphate dehydrogenase; *n* = 3/group; means + sem). *P < 0.05, **P < 0.01 compared with sham-treated mice; 1-way ANOVA.

To test whether such morphologic changes in glial cells are accompanied by actual glial cell activation, we measured the expression of various genes that are involved in glial cell activation after peripheral nerve injury, such as cathepsin S, *IRF8*, *IRF5*, and *Iba1*. Total RNAs were isolated from the dorsal horn of the spinal cord (L4–L5) 4 d after CCI, followed by quantitative real-time RT-PCR (**Fig. 9**). The RNA level of these microglial genes was increased in the ipsilateral dorsal horn after CCI, but was reduced in the pCK-HGF-X7–treated group. These results suggest that the control of microglia and astrocyte activation in the spinal cord dorsal horn might be a part of the pain control mechanism of pCK-HGF-X7 in the CCI model.

## DISCUSSION

HGF is an ideal candidate for the development of therapeutic agents that target the control of neuropathic pain and nerve regeneration, as it has been shown to possess potent angiogenic and neurotrophic properties. In the current study, we have demonstrated that an intramuscular injection of plasmid DNA that expresses both isoforms of HGF, pCK-HGF-X7, could effectively reduce neuropathic pain in a mouse CCI model. Two hundred micrograms of pCK-HGF-X7 in the mouse CCI model was demonstrated to be the optimum dose that generated a maximum pain-relieving effect. Expression levels of various injury- or pain-related genes, such as *ATF3*, *α2δ1*, and *CSF1*, were changed in a manner that was consistent with the analgesic effects of pCK-HGF-X7. Intramuscular injection of pCK-HGF-X7 also resulted in a decreased number of activated microglial cells and astrocytes, and down-regulation of related gene expression in the dorsal horn spinal cord. Taken together, our data strongly suggest that HGF produced from pCK-HGF-X7 might effectively reduce pain in a mouse CCI model by controlling the expression of pain-related genes.

Considering the fact that the pain-relieving effect of pCK-HGF-X7 was observed as early as 7 d after CCI and lasted up to 8 wk, the action points of this plasmid DNA might be divided in two. During the early stage, HGF protein released from transfected muscle cells may be directly involved in pain reduction by interacting with the c-Met receptor on sensory neurons to control pain- or injury-related genes by activating its downstream signaling pathways. In contrast, the long-term analgesic effect of pCK-HGF-X7 may be a result of the repair of the damaged nerve or the fundamental change in the pain circulatory in the CNS, as the expression of HGF from the plasmid DNA is no longer available at this late stage. Investigation of the underlying mechanism(s) are under way. Whatever the case, the pain-reducing effect observed after 2 wk of pCK-HGF-X7 injection should have resulted as a consequence of the biologic reactions that are triggered by exogenous HGF.

Data from the repeated-injection experiments have demonstrated that a second injection introduced 1 or 2 wk after the initial injection does not provide any additional effect on pain reduction; however, when the repeated injection was performed 3 or 4 wk later, the pain-relieving effect of pCK-HGF-X7 was further increased compared with that of the group that only received a single injection on the day of CCI. One possible explanation is that the HGF production from the initial injection ceases after 2 wk, and, therefore, a second injection administered only after this time point might be effective.

The dose response of pCK-HGF-X7 displayed an interesting bell- or U-shaped curve. The analgesic effect of pCK-HGF-X7 on neuropathic pain was increased only up to 200 μg, and at higher concentrations of DNA, it gradually diminished. This result is consistent with data from the actual phase I and II clinical studies involving pCK-HGF-X7 (VM202) that were conducted in patients with painful diabetic peripheral neuropathy (21, 22). In these clinical studies, 8 mg/leg of pCK-HGF-X7 produced a higher level of pain reduction than did 4 mg/leg, but 16 mg/leg was less effective than 8 mg/leg (21, 22). Although the reason for the bell- or U-shaped dose-response curve is not yet clear, our preliminary data suggest that the c-Met receptor might be degraded when the HGF level reaches beyond a certain point (unpublished data), as in the case of epidermal growth factor and epidermal growth factor receptor (57, 58).

ATF3, α2δ1, and CSF1 are all up-regulated when peripheral nerves are damaged in various ways, such as by sciatic nerve ligation or transection and CCI (41, 45–48); however, little has been known about how these genes are controlled in injured sensory neurons, but our results indicate that HGF may play a role by down-regulating the expression of these pain-related markers. Promoter sequence analysis predicted that the most common nucleotide sequence shared by ATF3, α2δ1, and CSF1 is the binding site for the activating protein 1 family. Activating protein 1 is a heterodimeric protein that long has been known to be involved in the development of neuropathic pain, dedifferentiation of Schwann cells, and regeneration of injured neurons (37, 59). It is also possible that HGF may somehow affect the expression of c-Fos or c-Jun and/or the activity of the DNA-binding protein; however, additional investigations are needed to understand of the role of HGF in the control of pain- and nerve injury–related factors.

Peripheral nerve injury or neuroinflammation lead to the development of neuropathic pain, which is characterized by the activation of glial cells, such as microglia and astrocytes. Microglial cells are known to undergo proliferation and activation during peripheral injury, releasing various cytokines and chemokines. They then act on respective cellular receptors expressed on astrocytes to stimulate these glial subtype cells (36, 55–56). Once astrocytes acquire reactive phenotypes, they are known to contribute to the maintenance of neuropathic pain (40, 60). Of interest, our results demonstrate a significant reduction in the number of activated microglia and astrocytes, which may explain the long-lasting pain-relieving effect of pCK-HGF-X7.

In summary, our results have demonstrated that an intramuscular injection of plasmid DNA that expresses HGF could produce sustained neuropathic pain relief in mice after CCI. Our data support the notion that HGF may exert neuroprotective effects *via* direct interaction with neurons and glia (61). Data from phase I and II studies for diabetic peripheral neuropathy demonstrated that pCK-HGF-X7 was safe and produces a remarkably high pain-reducing effect (22). In the current study, we have shown at least a part of the working mechanism of pCK-HGF-X7 in the alleviation of neuropathic pain. Taken together, pCK-HGF-X7 seems to have the potential to be a candidate for the next generation of therapeutic agents for the treatment of neuropathic pain.

## Supplementary Material

This article includes supplemental data. Please visit *http://www.fasebj.org* to obtain this information.

## Abbreviations

ATF3: activating transcription factor 3
CCI: chronic constriction injury
DRG: dorsal root ganglion
HGF: hepatocyte growth factor
hHGF: human hepatocyte growth factor
HPRT: hypoxanthine-guanine phosophoribosyltransferase
Iba1: ionized calcium-binding adapter molecule 1
IRF: interferon regulatory factor
TBST: Tris-buffered saline with Tween 20
