# Supplementary material for: Effective control of neuropathic pain by transient expression of hepatocyte growth factor in a mouse chronic constriction injury model
Source: FASEB J. 2018 Apr 16;32(9):5119–31. doi: 10.1096/fj.201800476R (PMC6113864; doi:10.1096/fj.201800476R)
Supplement: Supplementary file 1 [file fj.201800476R.sd1.docx]

**Supplementary Figure 1. I*.m.* injection of pCK-HGF-X7 ameliorates nerve injury-induced neuropathic pain for 8 weeks.** pCK and pCK-HGF-X7 (200 μg) were intramuscularly injected on the day of CCI, and the pain sensitivity towards mechanical stimuli was measured at appropriate times up to 8 weeks using von Frey filaments. Each group consisted of 6 mice, and more than 2 independent experiments were performed (mean + SEM; *, p<0.05; **, p<0.01; ***, p<0.001; one-way ANOVA). Sham-operated (■); CCI + pCK (**○**); CCI + pCK-HGF-X7 (●).

**Supplementary Figure 2. Baseline sensory function is not altered by i*.m.* injection of pCK or pCK-HGF-X7.** PBS, pCK, or pCK-HGF-X7 (200 μg) were intramuscularly introduced to naïve mice, and the effect of plasmid injection on baseline pain function was measured by **(A)** von Frey filaments and **(B)** Hargreaves tests (n = 6 for each group; one-way ANOVA).
