# Supplementary material for: Effective control of neuropathic pain by transient expression of hepatocyte growth factor in a mouse chronic constriction injury model
Source: FASEB J. 2018 Apr 16;32(9):5119–31. doi: 10.1096/fj.201800476R (PMC6113864; doi:10.1096/fj.201800476R)
Supplement: Supplementary file 2 [file fj.201800476R.sf1.pptx]

## Slide 1
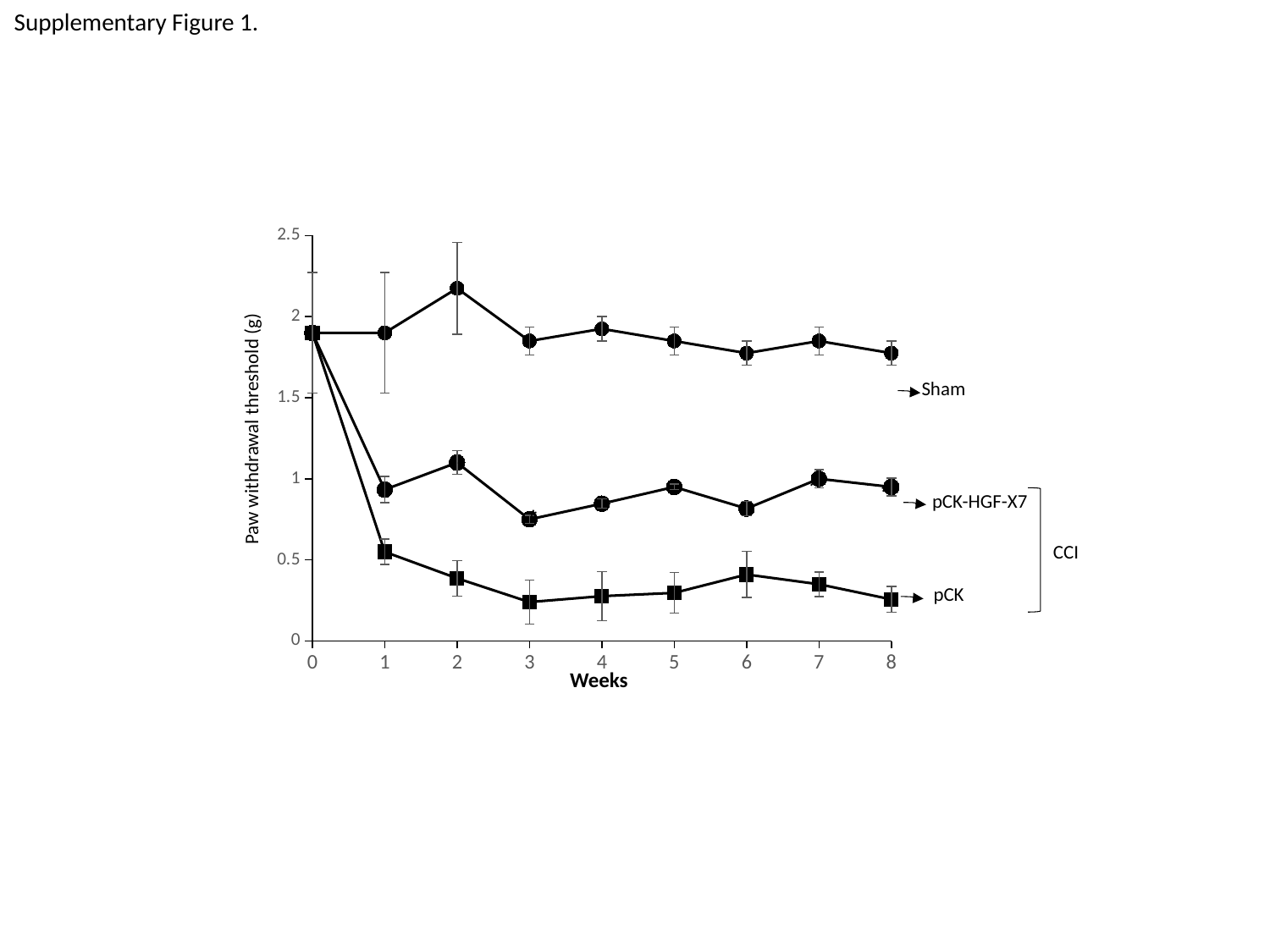

Supplementary Figure 1.
### Chart
| Category | | | |
|---|---|---|---|Sham
Paw withdrawal threshold (g)
pCK-HGF-X7
CCI
pCK
Weeks
*
*
*
*

## Slide 2
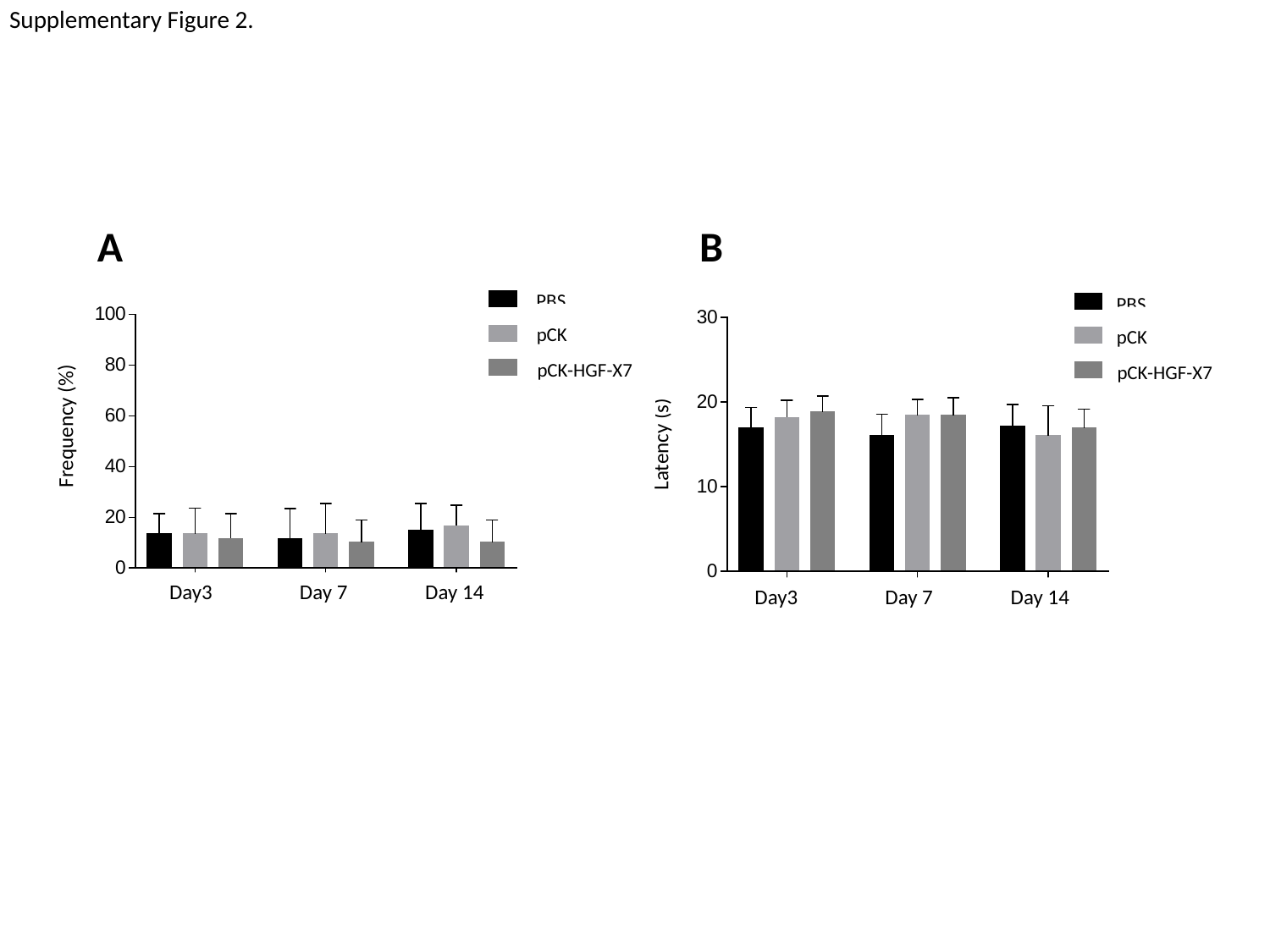

Supplementary Figure 2.
A
B
PBS
pCK
pCK-HGF-X7
PBS
PBS
pCK
pCK
pCK-HGF-X7
pCK-HGF-X7
 Frequency (%)
Latency (s)
Day3 Day 7 Day 14
Day3 Day 7 Day 14
